# Machine learning algorithms as new screening approach for patients with endometriosis

**DOI:** 10.1038/s41598-021-04637-2

**Published:** 2022-01-12

**Authors:** Sofiane Bendifallah, Anne Puchar, Stéphane Suisse, Léa Delbos, Mathieu Poilblanc, Philippe Descamps, Francois Golfier, Cyril Touboul, Yohann Dabi, Emile Daraï

**Affiliations:** 1 Department of Obstetrics and Reproductive Medicine, Hôpital Tenon, 4 rue de la Chine, 75020 Paris, France; 2 Clinical Research Group (GRC) Paris 6: Centre Expert Endométriose (C3E), Sorbonne University (GRC6 C3E SU), Paris, France; 3 Ziwig Health, 19 rue Reboud, Lyon, France; 4 Department of Obstetrics and Reproductive Medicine-CHU d’Angers, Angers, France; 5 Endometriosis Expert Center-Pays de la Loire, La Réunion, France; 6 Department of Obstetrics and Reproductive Medicine, Lyon South University Hospital, Lyon Civil Hospices, Bron, France; 7 Endometriosis Expert Center-Steering Center of the EndAURA Network, Paris, France; 8 Department of Surgical Oncology, Tenon University Hospital, 4 Rue de la Chine, 75020 Paris, France

**Keywords:** Medical research, Signs and symptoms, Mathematics and computing, Engineering, Biomedical engineering

## Abstract

Endometriosis—a systemic and chronic condition occurring in women of childbearing age—is a highly enigmatic disease with unresolved questions. While multiple biomarkers, genomic analysis, questionnaires, and imaging techniques have been advocated as screening and triage tests for endometriosis to replace diagnostic laparoscopy, none have been implemented routinely in clinical practice. We investigated the use of machine learning algorithms (MLA) in the diagnosis and screening of endometriosis based on 16 key clinical and patient-based symptom features. The sensitivity, specificity, F1-score and AUCs of the MLA to diagnose endometriosis in the training and validation sets varied from 0.82 to 1, 0–0.8, 0–0.88, 0.5–0.89, and from 0.91 to 0.95, 0.66–0.92, 0.77–0.92, respectively. Our data suggest that MLA could be a promising screening test for general practitioners, gynecologists, and other front-line health care providers. Introducing MLA in this setting represents a paradigm change in clinical practice as it could replace diagnostic laparoscopy. Furthermore, this patient-based screening tool empowers patients with endometriosis to self-identify potential symptoms and initiate dialogue with physicians about diagnosis and treatment, and hence contribute to shared decision making.

## Introduction

Endometriosis is defined as an inflammatory condition characterized by endometrial-like tissue outside the uterus^1,2^. The disease is estimated to affect 5–10% of women in the reproductive period, accounting for about 2.4 million women in France and approximately 190 million women worldwide^2,3^.

Endometriotic lesions can occur at different locations, including the pelvic peritoneum and the ovary, or infiltrate pelvic structures below the peritoneal surface (deep endometriosis)^2^. From a clinical point of view, endometriosis is a highly enigmatic condition with heterogeneous gynecological symptoms a source of systemic effects and impacting the social and psychological wellbeing of a woman, often resulting in decreased work performance^4–6^. In addition, symptoms may overlap with those of other common conditions (e.g., irritable bowel syndrome or interstitial cystitis), making differential diagnosis challenging ^7^.

Internationally, work is being undertaken to improve the awareness, diagnosis and treatment of endometriosis^8–11^. A global consortium of investigators in endometriosis recently published its recommendations for research priorities and highlights the challenges of developing a non-invasive screening tool to facilitate and improve diagnosis^9,12^.

In this specific setting, multiple biomarkers^13,14^, genomic analysis^15,16^, questionnaires^17–19^, symptom-based algorithms^17,20^, and imaging techniques^21^ have been advocated as screening and triage tests for endometriosis. However, none of them have been implemented routinely in clinical practice since none are of clinically relevant accuracy –defined by a sensitivity of 0.94 and a specificity of 0.79—to replace the direct visualization of lesions through laparoscopic surgery^13,14,21^.

Recent innovation in Artificial Intelligence (AI), Machine Learning (ML), and Deep learning (DL) is emerging as a promising statistical data-driven approach to solve a range of endemic issues, including for endometriosis^15,16,20,22,23^. In addition, wearable sensors^20,24,25^ and smartphones^26,27^ are being explored as a way of connecting medical researchers to patients, and vice versa. With these mobile technologies, patients can provide longitudinal, real-world evidence of their experience. For example, recent software platforms like ResearchKit (http://researchkit.org/) or Ziwig Health (https://ziwig.com/) facilitate the use of mobile technology and AI to recruit patients into studies.

We therefore designed a study (1) to train machine learning algorithms (MLA) to predict the likelihood of endometriosis, and (2) to validate MLA performance on unseen data from the Endo-mi RNA cohort study using the best performing trained models.

## Material and method

### Patient-generated data

The training dataset used in this study was pseudonymized data collected between January 2021 to May 2021 from the open health platform, Ziwig Health (https://ziwig.com/). This platform contains 8000 records of patients with symptom suggestive of endometriosis with 500 features about diagnosis, symptoms, imaging, medical treatment, fertility and surgical treatments, and follow-up. To create our training dataset to predict the likelihood of a diagnosis of endometriosis, we filtered the full Ziwig Health dataset to identify patient with diagnosis of endometriosis based on previous treatment for endometriosis or clinical examination confirming deep endometriosis, or sonography/MRI detecting ovarian, peritoneal or deep endometriosis. The control group was composed of patient with at least one symptom suggestive of endometriosis but without previous treatment for endometriosis or clinical examination confirming deep endometriosis, or sonography/MRI detecting ovarian, peritoneal or deep endometriosis. The training dataset included three types of data: numerical, categorical, and text. All the patients gave their consent to the use of their data in accordance with the data protection policy (RGPD), and in compliance with French law and the recommendations of the Commission Nationale de l'Informatique et des Libertés (CNIL). We obtained signed informed consent from all participants in the study. The experimental protocol was approved by le comité de protection des personnes (C.P.P.) Sud-Ouest et Outre-Mer 1 (CPP 1-20-095 ID 10476).

### Model training

#### Generality

**Figure 1:**
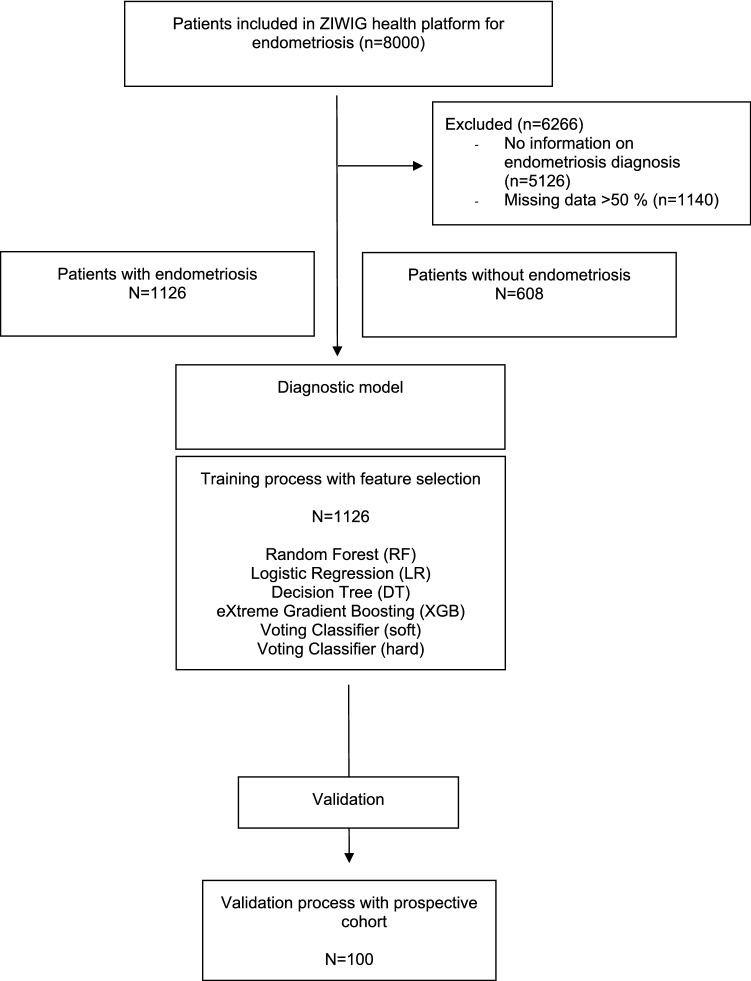
Flow chart of population for model development and validation.

Machine Learning, Deep Learning, and ensemble models are trained to developp a diagnostic tool for endometriosis. ML models such as Logistic Regression (LR), Random Forest (RF), Decision Tree (DT), eXtreme Gradient Boosting (XGB), and hard/soft Voting Classifier are considered ensemble learning techniques^28–34^. A flowchart of the training protocols employed in this study is detailed in Fig. 1.

### Model overview

- Logistic Regression (LR) is a statistical model that uses a logistic function to model a binary dependent variable. Mathematically, a binary logistic model has a dependent variable with two possible values, where the two values are labeled "0" and "1". Outputs with more than two values are modeled by multinomial logistic regression. Logistic Regression is used in various fields, including healthcare and social sciences^28^.
- Decision Tree (DT) is a simple and powerful machine learning model that utilizes any information obtained to find the best classification index of data samples. These classification indexes are the nodes of the DT, which then grow to form the tree structure. The DT model has already been successfully applied to research on public health and health behavior^29^.
- Random Forest (RF) classifier is an ensemble method that trains several DTs in parallel with bootstrapping followed by aggregation, jointly referred as bagging. Bootstrapping indicates that several individual DTs are trained in parallel on various subsets of a training dataset using different subsets of available features. Bootstrapping ensures that each individual DT in the RF is unique, which reduces the overall variance of the RF classifier. For the final decision, RF classifier aggregates the decisions of individual DTs and consequently exhibits good generalization^29^.
- eXtreme Gradient Boosting (XGB) is a gradient boosting algorithm which is an ensemble of weak prediction models, mostly DTs. An individual tree is a simple, often unreliable, model but when multiple trees are grouped together, they can create a robust algorithm. XGB starts by creating a simple tree, which than progresses sequentially and builds upon the weaker learners, with each iteration revising the previous tree until an optimal point is reached, such as the number of trees (estimators) to build the solution^34^.
- Voting Classifier algorithm is a machine learning model that trains on an ensemble of numerous models and predicts an output (class) based on their highest probability of a chosen class as the output. It simply aggregates the findings of each classifier passed into Voting Classifier and predicts the output class based on the highest majority of voting. Voting classifier supports two types of voting: hard voting where the predicted output class is a class with the highest majority of votes; soft voting where the output class is the prediction based on the average of probability given to that class^35^.
- Chi-Square Test: the Chi-square test is one of the most widely used non-parametric tests, often utilized to test the independence between observed and expected frequencies of one or more attributes in a contingency table. In this work, the Chi-square test was used to identify top significant features given the dependent variable (Y)^36^.

The performance of the MLAs was quantified with respect to sensitivity, specificity, F1-score, and discrimination criteria^37,38^.

### Model validation

The validation dataset was extracted from the prospective ENDOmiARN study (ClinicalTrials.gov Identifier: NCT04728152). The data of the women who participated in the study were aged between 18 and 43 years and had all undergone a laparoscopic procedure, either therapeutic laparoscopy for pain or infertility or diagnostic laparoscopic for chronic pelvic pain. Data collection and the analysis presented in this work were carried out under Research Protocol (n° ID RCB: 2020-A03297-32). For the aim of this study—to predict the likelihood of endometriosis diagnosis—the dataset contained 100 patient records after filtration. The accuracy of the MLAs was quantified with respect to sensitivity, specificity, F1-score, and discrimination criteria^37,38^.

## Results

### Epidemiological and surgical characteristics of the dataset

During the study period, 1126 patients and 608 were extracted from Ziwig Health platform (training set) with and without endometriosis to build the diagnostic model. In addition, 100 patients from the prospective cohort (validation set) have been used for the validation. All the patients included in both datasets had a surgical diagnosis of endometriosis. The general and clinical characteristics of the patients in the datasets are summarized in Tables 1 and 2. Significant differences in epidemiological features, symptom history, and medical therapies were found between the datasets.

**Table 1:** Demographic characteristics of the training dataset for patient with and without endometriosis.

	Patient with endometriosis N (%) = 1126	Patient without endometriosis N (%) N = 608	P < value
**Demographics characteristics**			
Age (mean ± SD)	29 ± 8	28 ± 9	< 0.001
BMI (body mass index) (mean ± SD)	23.41 ± 4.88	23.10 ± 4.56	0.12
Mother/daughter history of endometriosis			
Yes	21 (1.9%)	4 (0.7%)	
No	1105 (98.1%)	604 (99.3%)	0.056
**Endometriosis phenotype**			
Dysmenorrhea/VAS of Dysmenorrhea (mean ± SD)	6 ± 3.4	5 ± 3.2	< 0.001
Maximum length of periods (mean ± SD)	6 ± 4	5 ± 3	< 0.001
Abdominal pain outside menstruation			
Yes	721 (64.1%)	179 (29.4%)	< 0.001
No	405 (35.9%)	429 (70.6%)	
Pain suggesting sciatica			
Yes	427 (37.9%)	61 (10.1%)	
No	699 (62.1%)	547 (89.9%)	< 0.001
Pain on sexual intercourse	3.8 ± 3.5	2.3 ± 3.0	< 0.001
Lower back pain outside menstruation			
Yes	693 (61.5%)	200 (32.9%)	
No	433 (38.5%)	408 (67.1%)	< 0.001
Painful defecation (mean ± SD)	3.2 ± 3.3	1.5 ± 2.4	< 0.001
Alternating diarrhea/constipation during menstruation			
Yes	718 (63.7%)	234 (38.5%)	
No	408 (36.3%)	374 (61.5%)	< 0.001
Urinary pain during menstruation (mean ± SD)	1.4 ± 2.5	0.5 ± 1.4	< 0.001
Blood in the stools during menstruation			
Yes	179 (15.9%)	45 (7.4%)	< 0.001
No	947 (84.1%)	563 (92.6%)	
Blood in urine during menstruation			
Yes	150 (13.3%)	61 (10.1%)	
No	976 (86.7%)	547 (89.9%)	0.046
**Quality of life**			
Absenteeism duration in the last 6 months (mean ± SD)	7 ± 22	3 ± 12	< 0.001
Number of non-hormonal pain treatments used (mean ± SD)	1 ± 1	0 ± 1	< 0.001

**Table 2:** Demographic characteristics of the training and validation dataset.

	Training set N (%) = 1126	Validation set N (%) N = 100	P < value
**Demographics characteristics**			
Age (mean ± SD)	29 ± 8	31 ± 5	< 0.001
BMI (body mass index) (mean ± SD)	23.41 ± 4.88	24.3 ± 4.82	< 0.001
Mother/daughter history of endometriosis			
Yes	21 (1.9%)	8 (8%)	
No	1105 (98.1%)	92 (92%)	0.001
**Endometriosis phenotype**			
Dysmenorrhea/VAS of dysmenorrhea (mean ± SD)	6 ± 3.4	7.3 ± 3	< 0.001
Maximum length of periods (mean ± SD)	6 ± 4	8 ± 4	< 0.001
Abdominal pain outside menstruation			
Yes	721 (64.1%)	67 (67%)	
No	405 (35.9%)	33 (33%)	0.5527
Pain suggesting sciatica			
Yes	427 (37.9%)	53 (53%)	0.003
No	699 (62.1%)	47 (47%)	
Pain on sexual intercourse	3.8 ± 3.5	5.1 ± 3.5	< 0.001
Lower back pain outside menstruation			
Yes	693 (61.5)%	79 (79%)	0.00053
No	433 (38.5)%	21 (21%)	
Painful defecation (mean ± SD)	3.2 ± 3.3	4.2 ± 3.3	< 0.001
Alternating diarrhea/constipation during menstruation			
Yes	718 (63.7%)	80 (80%)	
No	408 (36.3%)	20 (20%)	0.0010
Urinary pain during menstruation (mean ± SD)	1.4 ± 2.5	1.9 ± 2.9	< 0.001
Blood in the stools during menstruation			
Yes	179 (15.9%)	20 (20%)	0.2862
No	947 (84.1%)	80 (80%)	
Blood in urine during menstruation			
Yes	150 (13.3%)	17 (17%)	0.3040
No	976 (86.7%)	83 (83%)	
**Quality of life**			
Absenteeism duration in the last 6 months (mean ± SD)	7 ± 22	23 ± 31	< 0.001
Number of non-hormonal pain treatments used (mean ± SD)	1 ± 1	2 ± 2	< 0.001

For the validation cohort, among those 100 women 87% (n = 87) were diagnosed with endometriosis and 13% (n = 13) without (controls). In both groups, the patients had pain symptoms suggestive of endometriosis. For the endometriosis patients, 51% (44/87) had rASRM stage I–II, and 49% (43/87) had stage III-IV. For all patients an MRI has been performed since this information was an inclusion criterion (https://clinicaltrials.gov/ct2/show/NCT04728152). Concerning the phenotype, among the 87 patients with endometriosis, we reported that 3% (n = 3/87), 6% (n = 5/87), 47% (n = 41/87), 44% (n = 38/87) had superficial endometriosis, endometrioma alone, deep infiltrating endometriosis alone, and both deep infiltrating endometriosis + endometrioma.

### Selection of significant features in the training set

#### Pre‐processing of dataset

The raw dataset contained 100 features some of which did not significantly affect the prediction of endometriosis occurrence. After taking suggestions from experts in endometriosis (SB, FG, PD, and ED), we selected a total of 16 essential clinical and symptom-based features related to history, demographics characteristics, endometriosis phenotype and treatment (Table 3) free available on the open health platform Ziwig. This approach gives a comprehensive analysis of results where models have been trained and validated on data. A flowchart of the training protocols employed in the study is detailed in Fig. 1.

**Table 3:** A summary of the 16 dataset features considered in the training approach.

**History**
Mother/daughter history of endometriosis
History of surgery for endometriosis
**Demographics characteristics**
Age
BMI (body mass index)
**Phenotype**
Dysmenorrhea/VAS of dysmenorrhea
Abdominal pain outside menstruation
Pain suggesting of sciatica
Pain during sexual intercourse
Lower back pain outside menstruation
Painful defecation
Urinary pain during menstruation
Right shoulder pain near or during menstruation
Blood in the stools during menstruation
Blood in urine during menstruation
**Quality of life**
Absenteeism duration in the last 6 months
**Treatment**
Number of non-hormonal pain treatments used

**Figure 2:**
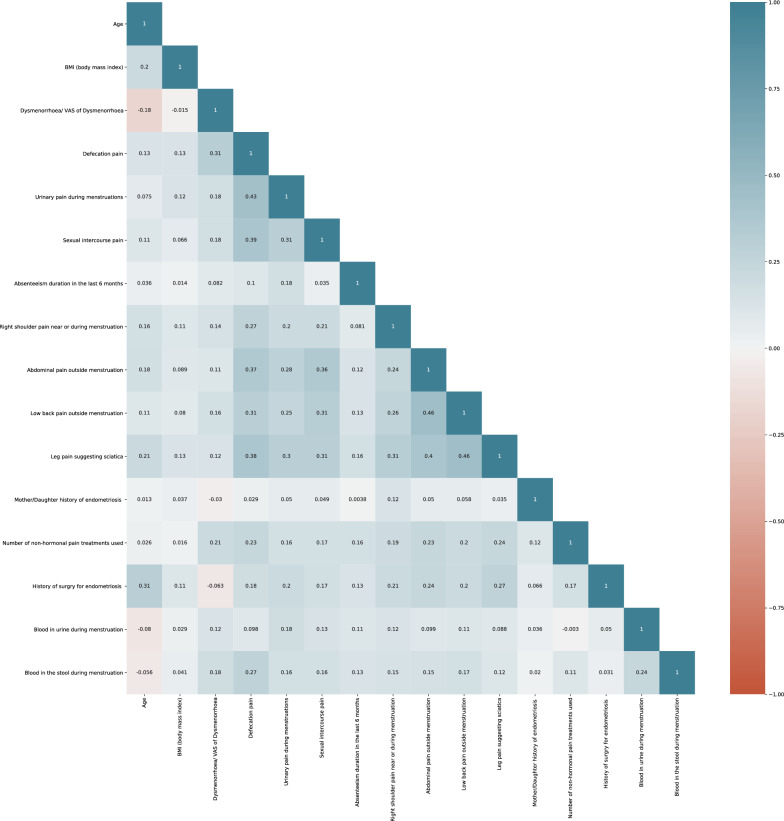
Correlation matrix of 16 features for the training set.

**Figure 3:**
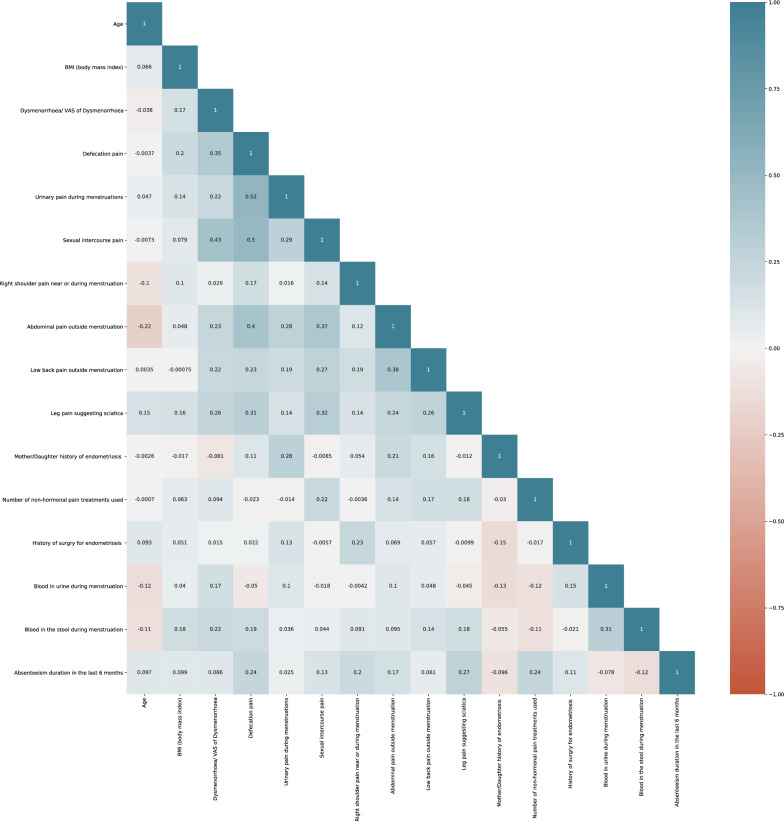
Correlation matrix of the 16 features for the validation set.

The top 16 features were used to train the ML model with RF, LR, DT, XGB, Voting Classifier (soft), and Voting Classifier (hard) algorithms (Table 4). A correlation matrix was constructed to reveal the importance of each of the features on the model developed (Figs. 2 and 3). Here we calculated the correlation coefficient between numerical and nominal columns as the Coefficient and the Pearson’s chi-square value^39^.

**Table 4:** Comparison between classification metrics of the different models in the training and validation sets.

Models	Training set				Validation set			
	Sensitivity	Specificity	F1-score	AUC	Sensitivity	Specificity	F1-score	AUC
Random forest (RF)	0.98	0.8	0.88	0.89	0.92	0.92	0.92	0.92
Logistic regression (LR)	1	0	0	0.5	0.95	0.81	0.87	0.88
Decision tree (DT)	0.82	0.8	0.81	0.82	0.91	0.66	0.77	0.78
eXtreme gradient boosting (XGB)	0.98	0.8	0.88	0.89	0.93	0.92	0.92	0.93
Voter classifier soft	0.98	0.6	0.74	0.75	0.93	0.88	0.9	0.90
Voter classifier hard	0.95	0.8	0.87	0.88	0.91	0.92	0.91	0.92

### Classification metrics of the training set

**Figure 4:**
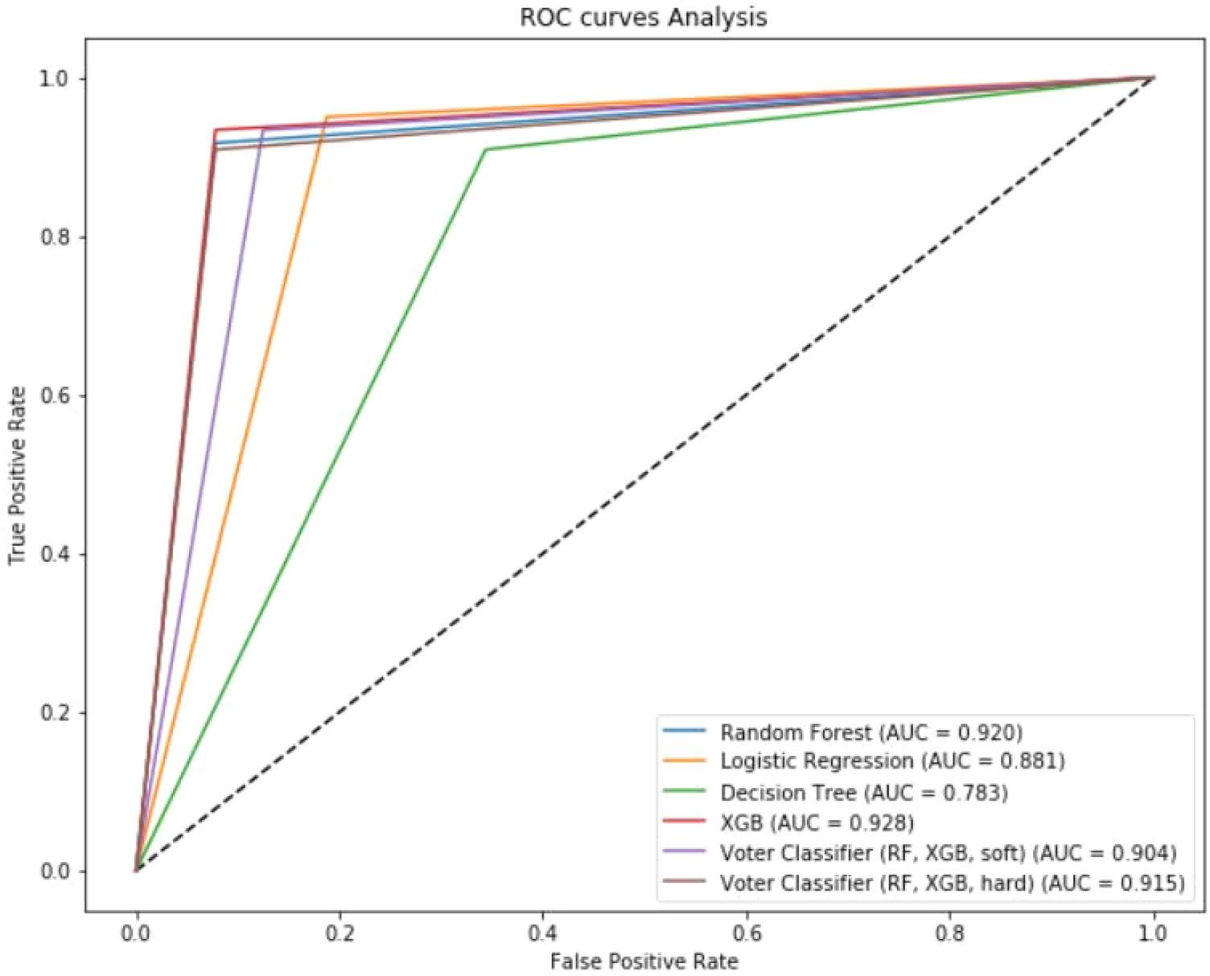
ROC curve analysis of models in training set.

The sensitivity, specificity, and F1-score of the 16 features for the MLA to diagnose endometriosis varied from 0.82 to 1, 0–0.8, 0–0.88, respectively. Table 4 summarizes the comparison between classification metrics of the different MLAs. Figure 4 summarizes the AUC-ROC curves in the training set.

### Classification metrics of validation set

**Figure 5:**
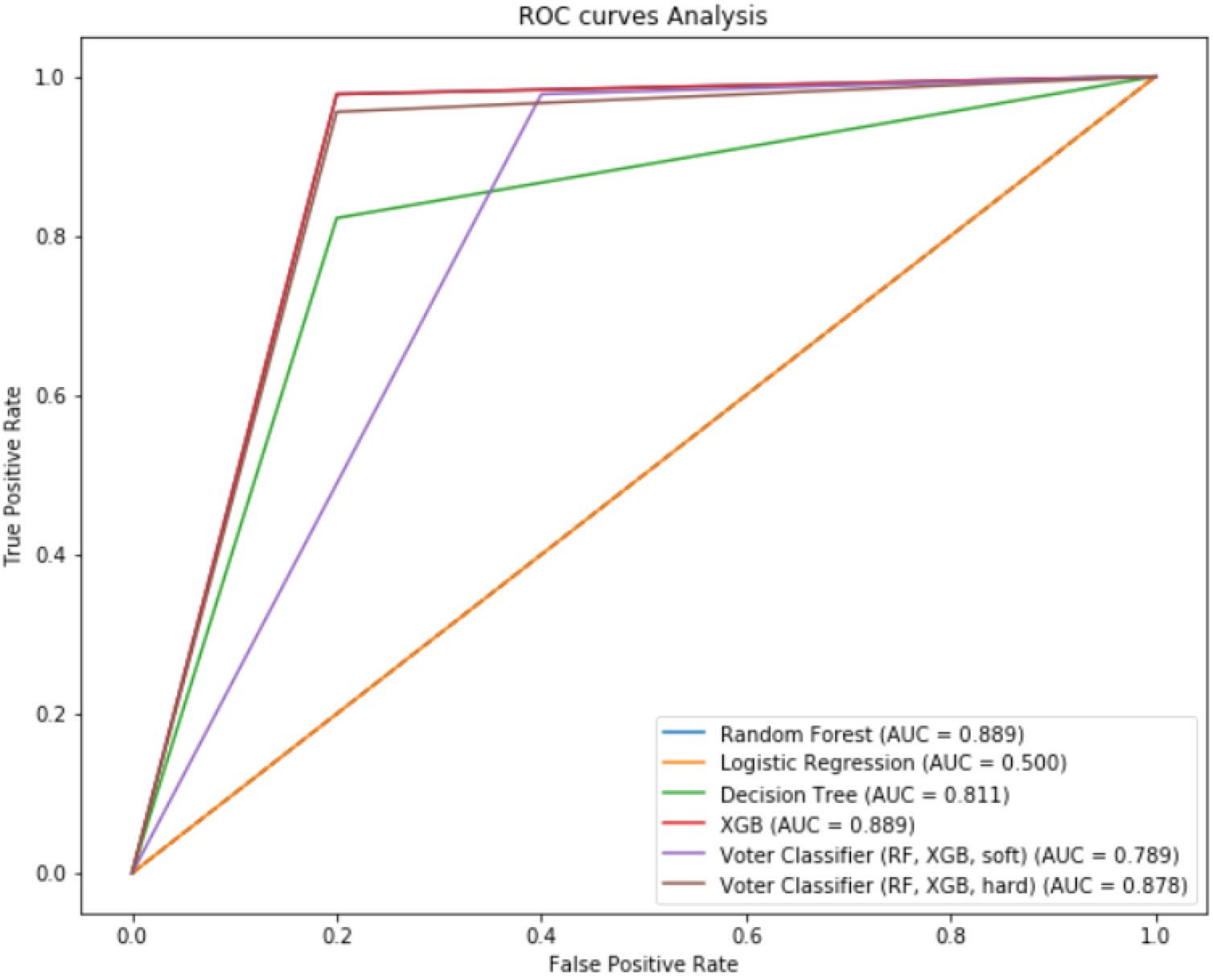
ROC curve analysis of different models in validation set.

The patient characteristics for the external validation set are summarized in Table 2. Significant differences were found between the patients’ phenotype profile compared with the training set. For the 16 most important features selected, the sensitivity, specificity, and F1-score varied from 0.91 to 0.95, 0.66–0.92, 0.77–0.92, respectively (Table 4). Figure 5 summarizes the AUC-ROC curves in the validation set.

## Discussion

The present study demonstrates that MLAs based on 16 clinical and symptom-based features enables diagnosis and early prediction of endometriosis onset. The resulting metrics of the model supports the clinical interest of this tool as a screening test for general practitioners (GPs), gynecologists, and other front-line healthcare providers. Patients could also use this tool themselves and it may reduce “diagnostic wandering”, and hence diagnostic delay, and result in earlier treatment.

The comparison between the models’ metrics supports the clinical value of MLAs as a screening tool to improve the endometriosis patient care pathway with a sensitivity and specificity of 95% and 80%, respectively. This is in agreement with the Cochrane review of Nisenblat et al.^14^ underlining that the predetermined criteria for a clinically useful non-invasive test to replace diagnostic laparoscopy were a sensitivity and specificity of 0.94 and 0.79, respectively. Using AI, we confirmed the value of MLA tools with an external validation study on a very different population in terms of endometriosis phenotypes and patient characteristics, suggesting its reproducibility and accuracy. In this specific setting, few data are available on the contribution of AI for the diagnosis and triage of endometriosis. Recently, Kleczyk et al.^23^ validated the role of MLAs for the diagnosis, prediction, and forecasting of endometriosis, based on a medico-economic healthcare database. However, although accurate from a statistical point of view, the clinical utility of this tool is questionable because of (1) the inclusion in the models of key features often associated with other gynecologic disorders such as pelvic inflammatory, sub-mucous myoma or genital infection, (2) the lack of a digital personalized patient-based approach^17,40^, and (3) the lack of external validation to assess its reproducibility. The present MLA tool is a complete patient-based screening questionnaire in accordance with the recent NHS England guidance on patient involvement in their health and care, by which they mean “supporting them to become involved, as much as they want or are able to, in decisions about their care and giving them choice and control”^40^. It supports the use of self- management approaches that reenforce patients as experts in their own health and provides support to develop understanding and confidence, improved patient experience and adherence to treatment and medication^17,25,27,31,32,40^.

In the last decade, strategies to advance precision medicine have attracted considerable investment in developing new diagnostic methods, treatments, and disease prevention initiatives^15,19,26,32,41,42^. Virtual medical assistants using AI have recently matured and are being used in various health settings^15,20,25,30,43^. In the current study, our MLA screening questionnaire is associated with a sensitivity, specificity, F1-score, and AUC ranging from 0.82 to 1, 0–0.8, 0–88, and 0.5–0.89 in the training and validation sets based on the combination of 16 key common criteria. Interestingly, most of the features included in the MLAs are related to the patient’s history, clinical phenotype, and impact on quality of life. Among the MLAs, Soft Voting Classifier, RF and XGB appear the most accurate methods with a sensitivity and specificity ranging between 95 and 98% and 80%, respectively. Similarly, Yeung et al. developed a predictive model for early endometriosis stages based on a preoperative questionnaire. The model was able to differentiate women with endometriosis from those without (AUC = 0.822, P < 0.001; sensitivity = 80.5%; and specificity = 57.7%); however, the specificity is low and it cannot be used as a simple self-completed measure given its complex scoring^44^. In this setting, the scoping review from Surrey et al.^17^ concerning symptom-based screening tools for endometriosis highlighted that only one study evaluated a questionnaire that was solely patient-completed, and that most of the others reported hybrid measures consisting of patient-completed, clinician-completed, imaging, and/or laboratory-based assessments to predict diagnosis.

The strength of the present study is the use of web-based diagnostic tools and symptom checkers that may increase patient health literacy and promote proactive health-seeking behavior. Our diagnostic tool is easily accessible and free for both patients and healthcare providers^20,24,26,27^. Previous studies have underlined the medical contribution of a low-cost method of self-management for healthcare providing effective motivation, and may potentially avoid negative experiences associated with interacting with a health professional who may be perceived as patronizing, judgmental or non-supportive^45,46^. This is especially relevant for endometriosis. Digital interventions may be particularly useful in supporting disadvantaged populations, and particularly adolescents, because user experience less stigmatizing than conventional strategies^47^. Finally, with mobile technologies, patients can provide longitudinal, real-world evidence of their experience. This is of particular relevance for patients seeking to confirm a diagnosis of endometriosis. In a large cohort study, Ballweg et al.^48^ reported that, among patients with symptoms suggestive of endometriosis, 61% of the healthcare professionals said there was “nothing wrong” contributing to a delay in diagnosis. This was confirmed by Greene et al.^49^ who showed that time from onset of symptoms to seeking medical attention and time from seeking medical attention to diagnosis were 4.6 years and 4.7 years, respectively, irrespective of the healthcare provider involved. Hence, the contribution of AI could be crucial as it offers objective data which will improve awareness of endometriosis among healthcare professionals with direct consequences on diagnostic and therapeutic management and the possible referral of patients to expert centers.

In a review of the literature on endometriosis, Zondervan et al.^2^ underlined the low contribution of specific questionnaires as a triage test to diagnose endometriosis. Moreover, clinical examination as well as transvaginal sonography (TVUS) are not always acceptable particularly for adolescents and virgin patients. Bazot et al.^50^ demonstrated that diagnosis of deep endometriosis or endometriomas is easy using TVUS or MRI. However, the meta-analysis of Nisenblat et al.^21^ demonstrated that although diagnosis by TVUS or MRI was accurate for rectal endometriosis and pouch of Douglas obliteration, fulfilling the criteria for SpIN triage tests, imaging techniques were less accurate for other lesions such as utero-sacral ligament endometriosis which is the most frequent location of deep endometriosis. Moreover, imaging techniques have a low accuracy for detecting peritoneal endometriosis which represents the earlier stage of the disease^21^. Conversely, our laparoscopic data demonstrated that AI alone offers a high accuracy for diagnosing endometriosis even in patients with early disease stage which raises the question of the relevance of diagnostic laparoscopy. Although specialized centers with multidisciplinary teams will surely remain part of the care pathway, particularly for referral from GPs, AI could resolve screening, triaging and assessment issues and help patients navigate the healthcare system which is currently a major concern.

Despite the high accuracy of AI for diagnosing endometriosis, some limitations of the present study deserve to be underlined. First, our population was based on self-questionnaire available on the platform including a large number of items not always fulfilled by the patients with a number of patient with > 50% at 1140 on 8000. Moreover, the patient was asked whether there are or not endometriosis with a potential bias in the control group. Indeed, it has been demonstrated that endometriosis could be asymptomatic in up to 20% of patients^21^. This reinforces the concept of objective test to diagnose endometriosis. Nisenblat et al. underlined that no biomarker of combination of biomarkers can accurately assess the diagnosis of endometriosis^21^. However, a recent study Moustafa et al., suggested the relevance of blood signature of endometriosis based on a limited number of mi RNA, raising the issue to reflect the heterogeneity of endometriosis^51^. This is also underline by Vahnie et al., showing that even using 42 mi RNA no models achieve the value for a SNoUT test^14,52^. Second, the validation set was composed of a relatively small sample size which cannot rule out all potential biases. However, this population was homogeneous and corresponded to patients with suggestive symptoms of endometriosis and having undergone systematic diagnosis of severe endometriosis forms by imaging techniques with surgical confirmation. In this specific setting, Nisenblat et al. demonstrated that imaging techniques for rectal endometriosis had a sensitivity of 0.96 (95% CI 0.86–0.99) and a specificity of 0.98 (95% CI 0.94–1.00), a sensitivity of 0.87 (95% CI 0.69–0.96) and a specificity of 0.98 (95% CI 0.95–1.00) for obliterated pouch of Douglas, a sensitivity of 0.82 (95% CI 0.60–0.95) and a specificity of 0.99 (95% CI 0.97–1.0) for vaginal wall endometriosis, and a sensitivity of 0.88 (95% CI 0.47–1.0) and a specificity of 0.99 (95% CI 0.96–1.0) for rectovaginal septum endometriosis, thus fulfilling the criteria for SpIN triage tests^21^. Moreover, all the patients with early disease stages, who represent a crucial challenge, underwent a diagnostic laparoscopy with systematic biopsy. A second limitation is the absence of patients with discordant features such as symptoms suggestive of endometriosis with negative clinical examination and MRI in the validation set.

In conclusion, our data support the use of MLAs to diagnose endometriosis thereby questioning the relevance of diagnostic laparoscopy and thus constituting a real paradigm change in clinical practice^2,13,14^. Since delays in diagnosis may contribute to undertreatment, continued pain, and prolonged symptom impact which impairs women’s quality of life, helping patients to recognize their symptoms is a crucial step toward diagnosis and effective management of endometriosis. Patient-based screening tools empower patients with endometriosis to self-identify potential symptoms and initiate dialogue with physicians about diagnosis and treatment hence contributing to shared decision making.
